# Electrically turning periodic structures in cholesteric layer with conical–planar boundary conditions

**DOI:** 10.1038/s41598-021-87854-z

**Published:** 2021-04-16

**Authors:** Oxana Prishchepa, Mikhail Krakhalev, Vladimir Rudyak, Vitaly Sutormin, Victor Zyryanov

**Affiliations:** 1 Kirensky Institute of Physics, Federal Research Center KSC SB RAS, Krasnoyarsk, Russia 660036; 2 Institute of Engineering Physics and Radio Electronics, Siberian Federal University, Krasnoyarsk, Russia 660049; 3 Faculty of Physics, Lomonosov Moscow State University, Moscow, Russia 119991

**Keywords:** Liquid crystals, Liquid crystals

## Abstract

Electro-optical cell based on the cholesteric liquid crystal is studied with unique combination of the boundary conditions: conical anchoring on the one substrate and planar anchoring on another one. Periodic structures in cholesteric layer and their transformation under applied electric field are considered by polarizing optical microscopy, the experimental findings are supported by the data of the calculations performed using the extended Frank elastic continuum approach. Such structures are the set of alternating over- and under-twisted defect lines whose azimuthal director angles differ by $180^\circ$. The $U^+$ and $U^-$-defects of periodicity, which are the smooth transition between the defect lines, are observed at the edge of electrode area. The growth direction of defect lines forming a diffraction grating can be controlled by applying a voltage in the range of $0\le \, V \le 1.3$ V during the process. Resulting orientation and distance between the lines don’t change under voltage. However, at $V>1.3$ V $U^+$-defects move along the defect lines away from the electrode edges, and, finally, the grating lines collapse at the cell’s center. These results open a way for the use of such cholesteric material in applications with periodic defect structures where a periodicity, orientation, and configuration of defects should be adjusted.

## Introduction

Cholesteric liquid crystals (CLCs) form helicoidal orientational structure at equilibrium, which results in a variety of possible director configurations and corresponding unique optical properties of materials based on them^1–5^. It allows using CLCs in controllable reflectors^6,7^, shutters^8–10^, diffraction and holographic gratings^11–15^, tunable lasers^16,17^, etc. The forming structure is determined by the boundary conditions (surface anchoring), CLC material properties, ratio between layer thickness *d* and equilibrium helix pitch $p_0$. Up to date, the structures formed by CLCs under planar (tangential), homeotropic and homeo-planar surface anchoring have been thoroughly studied. In the case of tangential anchoring, homogeneous planar twisted structure, domain structure, and planar structure with oil strips defects were found^3,4,18–20^. At homeotropic anchoring, structures with multiple soliton-like defects (the bubbles, hopfions with the different Hopf index values, strips)^21,22^ and fingerprint textures^23^ were obtained. In the case of hybrid homeo-planar boundary conditions (homeotropic anchoring on the one substrate and planar anchoring on another one), the defect-free twisted structure or the modulated periodic director configuration are formed depending on the value of ratio $d/p_0$^24–28^.

One of the cornerstones for research and application of CLCs is to prepare the uniformly twisted and periodic structures and to switch between them. Under tangential anchoring, the aim is to switch between the uniformly twisted configuration (reflects light) and domain structure (strongly scatters light)^29^. At homeotropic anchoring, the azimuthal degeneration of director orientation occurs, which results in random mutual orientation of the strips and soliton-like defects.

Typically, external action or special substrate preparation are used to obtain periodic structures. For instance, aligned bubbles can be obtained using laser radiation^30^ or substrates with a specific profile^31,32^. Such substrates contain orienting coating, which forms periodically located bands with homeotropic or planar CLC anchoring enforcing the formation of strip structure^33,34^. Preparation of the periodic planar anchoring on the substrate with the period similar to the equilibrium helix pitch results in the uniformly twisted structure, whose helicoid axis deviates from substrate plane by an intermediate angle between 0$^\circ$ and 90$^\circ$^35,36^. In the case of homeo-planar anchoring, azimuthal degeneration of the forming periodic structure is avoided due to the presence of the substrate with homogeneous planar anchoring. As a result, the orientation of modulated periodic structure is specified by the ratio $d/p_0$. This structure can be controlled effectively by adjusting $p_0$ (for example, with photosensitive chiral dopant)^28^.

The cholesteric structures under conical or conical-planar boundary conditions are less studied at the moment. At conical anchoring, director on substrate is oriented at the polar angle $0^\circ< \theta _0 < 90^\circ$ and it has azimuthal degeneration. Hybrid conical-planar anchoring can be formed under the wetting phenomenon. In this case, defectless nematic-like structure or periodic structure are formed depending on the CLC layer thickness^37^. Earlier, we have studied the orientational structures of nematic and cholesteric LCs at conical-planar anchoring with tilt angle $\theta _0 \cong 50^\circ$ assigned by the polymer film^38^. It has been shown that the defectless twisted structure, the structures with defect loops or the pair of over-twisted and under-twisted defect lines are formed depending on the thickness CLC layer *d* and the ratio $d/p_0$. The effect of electric field on these structures has been considered in ref.^39^. It has been demonstrated, that the twist angle of CLC structure decreases under increasing electric field, which causes instability of the defects. As a result, the defect loop shrinks and collapses, and the over-twisted defect line can transform into the third defect type. However, the influence of electric field on the CLC periodic structure formed under conical-planar anchoring was not studied. In this work we examine the action of an electric field perpendicular to the cell plane by both the experiment and computer simulations, consider possible orientations of periodic structure relative to the rubbing direction as well as the modes of their transformation at various voltages and the relaxation process. Comparing the results of both methods, we make conclusions about the features of the observed response of the CLC layer.

## Results

**Figure 1:**
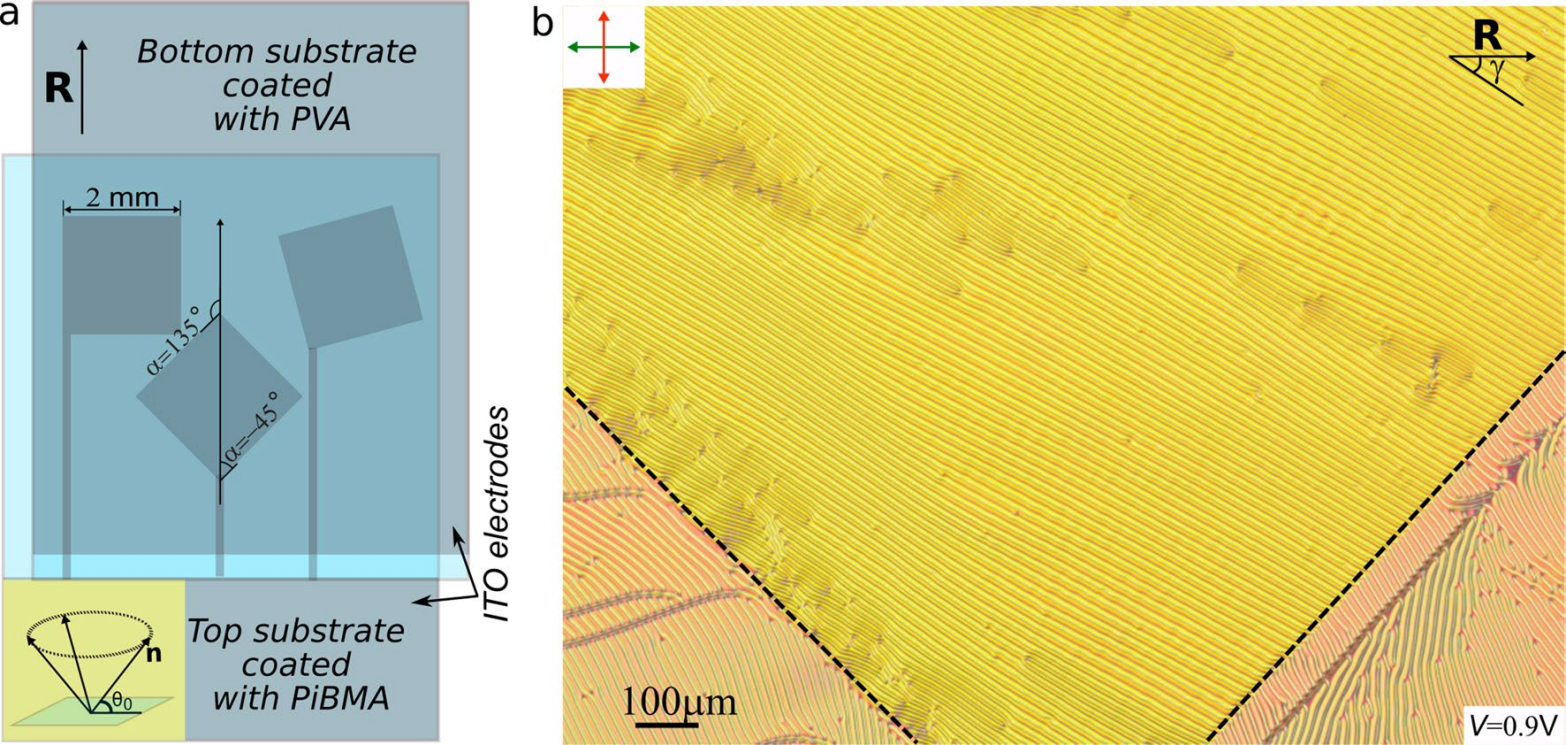
Scheme of the experimental cell in which the electrically induced transformation of CLC is studied inside the square areas of ITO electrodes oriented at different $\alpha$ angles to the rubbing direction (**a**). The square side is 2 mm. The conical anchoring with the tilt angle $\theta _0 =50^\circ$ is schematically shown in the insertion. Polarizing optical microscopy image of the cell area under the voltage *V* = 0.9 V (**b**). Two borders of ITO electrode (black dashed lines) are oriented at angles $\alpha = -45~^\circ$ and $\alpha = -135~^\circ$ to the rubbing direction **R**. $\gamma$ is an angle between the rubbing direction **R** and strips. Hereinafter the rubbing direction **R** of PVA film is shown by the single black arrow. The double arrows indicate the polarizer (green arrow) and analyzer (red arrow) orientations.

We have considered CLC-filled sandwich-like cells of thickness $d = 5.0~\upmu$m consisting of two glass substrates with transparent ITO electrodes coated with polymer films (Fig. 1a). The CLC consisted of nematic mixture LN-396 and cholesteryl acetate (characteristic ratio $d/p_0 = 0.6$, see "Methods" section for details).

The bottom substrate was covered by polyvinyl alcohol (PVA) film and then it was unidirectionally rubbed specifying homogeneous planar anchoring. The top substrate was covered by the poly(isobutyl methacrylate) (PiBMA) film without additional treatment after the deposition process, which results in conical boundary conditions with the tilt angle $\theta _0 \cong 50^\circ$^40,41^. The polymer films were deposited on the substrates by spin coating method. ITO layer on the top substrate was etched in the form of the square of $2 \times 2$ $\hbox {mm}^2$ with borders oriented at different angles $\alpha$ to the rubbing direction $\mathbf {R}$ (Fig. 1a).

For detailed structure analysis, we performed calculations of CLC structure within a layer of thickness *d* filled with chiral nematic by the extended Frank elastic continuum approach (see "Calculations of layer structure" section for details). We used cuboid simulation box with periodic boundary conditions over *x* and *y* dimensions. The bottom surface was set with strong planar aligned boundaries ($\mu _1 = W_1d/K_{11} = 1000$, $\theta _0=0$). The top surface had weak conical boundary conditions ($\mu _2 = W_2d/K_{11} = 40$, $\theta _0=50^\circ$). The equilibrium characteristic ratio $d/p_0=0.6$ was set according to the experimental data. First, we calculated the equilibrium structure at no electric field by full energy optimization for all possible angles $\gamma$ between the rubbing direction **R** and periodic strips (Fig. 1). Second, we simulated the electrically induced transformation of CLC structure by relaxation after field switching on or off. The data below are shown in dimensionless electric field units $e=|{\mathbf {E}}|{d}\left( \varepsilon _0\Delta \varepsilon /K_{11}\right) ^{1/2}$.

### Equilibrium orientational structure

**Figure 2:**
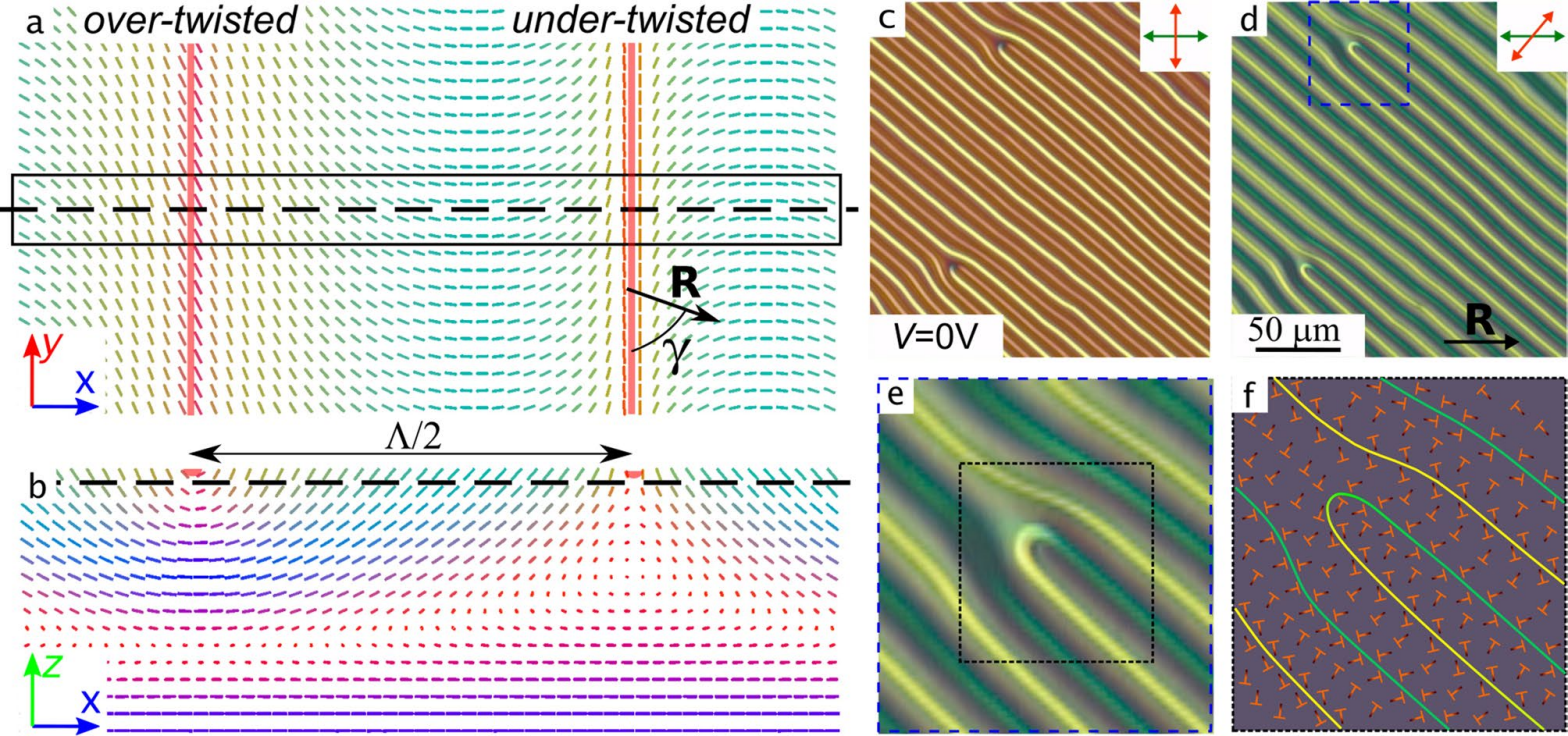
Energy-optimal simulated structure of cholesteric layer: top view, cross-cut at the top surface (**a**); side view, cross-cut perpendicular to the strip lines (**b**). Dashed black lines show cross-cut directions. The director vectors are colored in correspondence with their direction (*x*—blue, *y*—red, *z*—green). POM images of CLC layer taken in the crossed polarizers (**c**) and at angle $40^\circ$ between polarizer and analyzer (**d**) when $V=0$ V. The enlarged area with $\hbox {U}^+$-defect marked by the blue frame in Fig. 2d (**e**). The scheme of director distribution on the top substrate near $\hbox {U}^+$-defect (**f**).

The type of forming structure in the studied system under conical-planar boundary conditions (the defectless twisted structure, the structure with defect lines or periodic structure) depends on both the ratio $d/p_0$ and the CLC layer thickness^38^. It is caused by a weak surface anchoring strength ($W_s$~$10^{-6}$ J $\hbox {m}^{-2}$) realized for conical anchoring. For the cell under study with $d/p_0=0.6$ and $d=5\,\upmu$m thickness, the periodic structure of strips is formed (Fig. 1b). It should be emphasized, initially, when the cell is filled by CLC we observe randomly distributed areas with the strips oriented at various angles in the range of $40^\circ \lesssim \gamma \lesssim 100^\circ$ to the rubbing direction (see Supplementary Figure S1 and Fig. 1b). Frequently, the adjacent areas are divided by the sharp border where a multitude of periodically arranged defects (U-defect) get formed. The same defects of periodicity appear in the areas with uniform orientation of strips. For instance, the area with angle $\gamma =40^\circ$ containing two such U-defects is presented in Fig. 2c,d.

We have calculated the director distribution corresponding to the ideal periodic system with strips (Fig. 2a,b and Supplementary Figure S2). To find energy-optimal angle $\gamma$, we calculated structures for various lengths of the periodic box $2d\le L_x \le 4d$ and all possible angles $\gamma$. The system with the minimum free energy showed the optimal period $\Lambda _{calc} = (3.3\pm 0.1)d=(16.5\pm 0.5)\,\upmu$m between the strips and simultaneously the optimal angle $\gamma =70^\circ$. The black box in Fig. 2a shows actual simulation box, while the whole image is multiplied over *y* direction via periodic boundaries for easier understanding. The main feature of the structure is the pair of parallel surface defect lines located on the substrate with conical anchoring. These are virtual defects, i.e. containing no singularity but surface boundary mismatch, where the director tilt angle changes to the opposite one. Figure 2b demonstrates side view, the cross-cut perpendicular to the strips lines. One can see in Fig. 2a, b that the director has larger azimuthal angle near one line (over-twisted defect line), and it has smaller azimuthal angle near another one (under-twisted defect line). The difference between the azimuthal twist angle of director $\varphi _d$ on the substrate with conical anchoring for over-twisted defect and under-twisted defect lines equals to $\pi$ (Fig. 2a, see Supplementary Figure S2). The twist director angle specifies an ellipticity and azimuth of light polarization passed through the twisted structure^42,43^.

In the experiment, different azimuthal twist director angles cause various colors of over- and under-twisted defect lines observed in the crossed polarizers (Fig. 2c). Thus, the maximum color contrast between the defect lines is observed when the polarizer is parallel (perpendicular) to the director at the bottom substrate and the analyzer is oriented perpendicular (parallel) to the defect lines, i.e. the director at the top substrate (Fig. 2d, e). When the CLC cell is not affected by external factors, the orientational structure in it remains unchanged for a long time.

The above-mentioned imperfections in the periodic structure are smooth transition between over-twisted and under-twisted lines by 180$^\circ$ turn of the defect line ($\hbox {U}^+$-defect, see Fig. 2c–f). First, $\hbox {U}^+$-defects (Fig. 2e) allow to distinguish easily over- and under-twisted lines from each other. Also, they play an important role in the response and relaxation of periodic structure on an electric field. In the experimental setup, the cell is filled by the left-handed cholesteric and illuminated from the side of the bottom substrate with planar anchoring. Thus, yellow lines in the POM images (Fig. 2d,e) correspond to the smaller $\varphi _d$ angle (under-twisted defect lines), while green lines correspond to the larger twist angle (over-twisted defect lines). The corresponding director distribution near the top surface with conical anchoring is shown in Fig. 2f.

**Figure 3:**
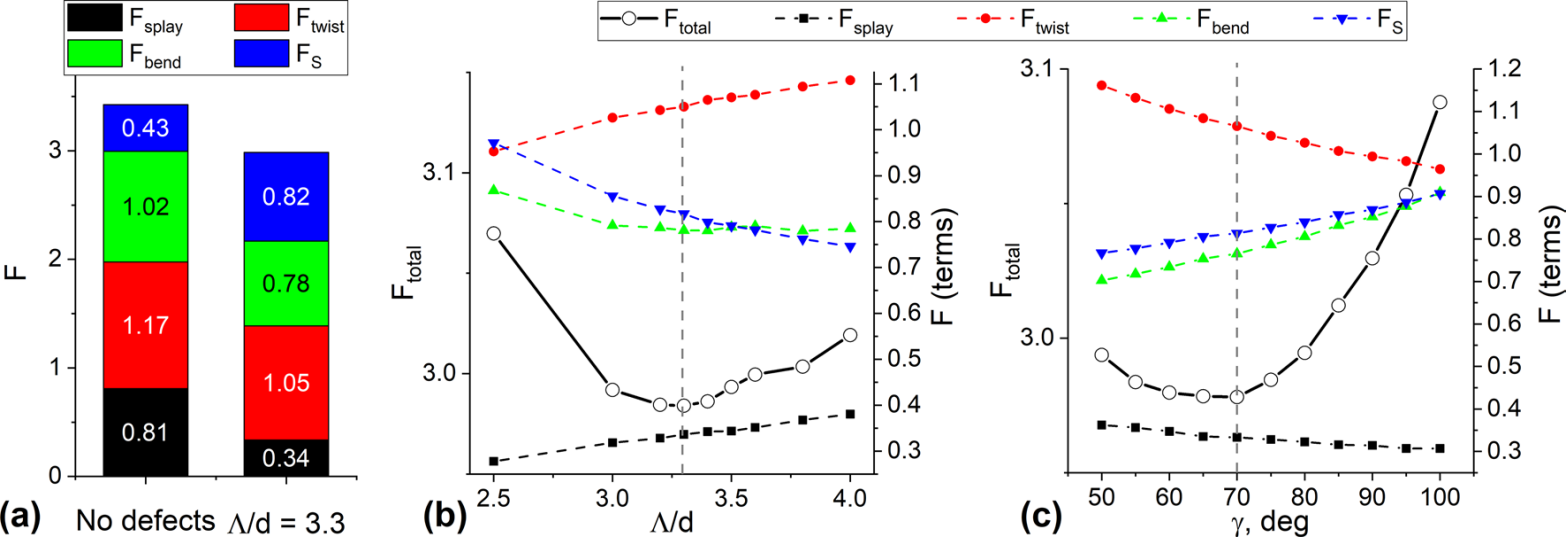
Total free energy per $d^2$ film surface and its terms of the calculated structures: comparison of energy terms for defectless structure and the optimal one with periodic defect lines (**a**), free energy for periodic structure with defects: dependence on normalized period $\Lambda /d$ for $\gamma =70^\circ$ (**b**), and dependence on $\gamma$ for $\Lambda /d=3.3$.

Computer simulations give a detailed explanation of obtained periodic structure. First of all, let us compare the energies of the two states in the absence of an electric field: the one with no defects and other with periodic defect lines of energy-optimal period (Fig. 3a). The state with periodic defect lines has lower total free energy and thus is stable. The reasons are very simple: while the surface energy is higher in this state in comparison to one with no defects, the elastic energy is significantly lower, especially the energy of splay deformations. In the defectless structure, the upper half of the layer (close to the conical boundaries) exhibits high splay and bend deformations (presumably, in order to minimize the twist term). At the same time, the structure with periodic surface defects has splay and bend deformations only near the defect lines (mostly near the ones shown in green in Fig. 2d–f). Since the dimensionless anchoring strength $\mu =Wd/K_{11}$, then type of formed structure (defect or defectless) depends on the thickness layer *d*. So, at the same $d/p_0$ the periodic structure is formed within thin layer while the defectless structure is formed in the thick sample^38^. The mechanism determining the value of the strip period of the defect structure also can be understood from the energy balance point of view. In Fig. 3b, free energy per $d^2$ surface area and its terms are shown as a functions of $\Lambda /d$ at fixed $\gamma =70^\circ$. From one side, both surface anchoring and bend energies drive the system to a higher distance between the strips. The reason is pretty clear: the lower the defect density, the lower the energies originating from it. At the same time, splay and twist energies drive the system the opposite way (in the region shown on these plots), because higher density of defect lines allows the system to fit the intrinsic twist power of the cholesteric LC. The energy minimum found in experiment and computer simulations represents the optimal point for the sum of these two factors. The optimal value of the angle $\gamma$ (rotations of the defect lines in correspondence to the rubbing direction) originates from the same type of balance (Fig. 3c). Splay and twist deformations force the system to increase the $\gamma$ angle, surface anchoring and bend deformations force the system to decrease it, and the outcome is the equilibrium around 70$^\circ$.

### The periodic structure response on electric field

**Figure 4:**
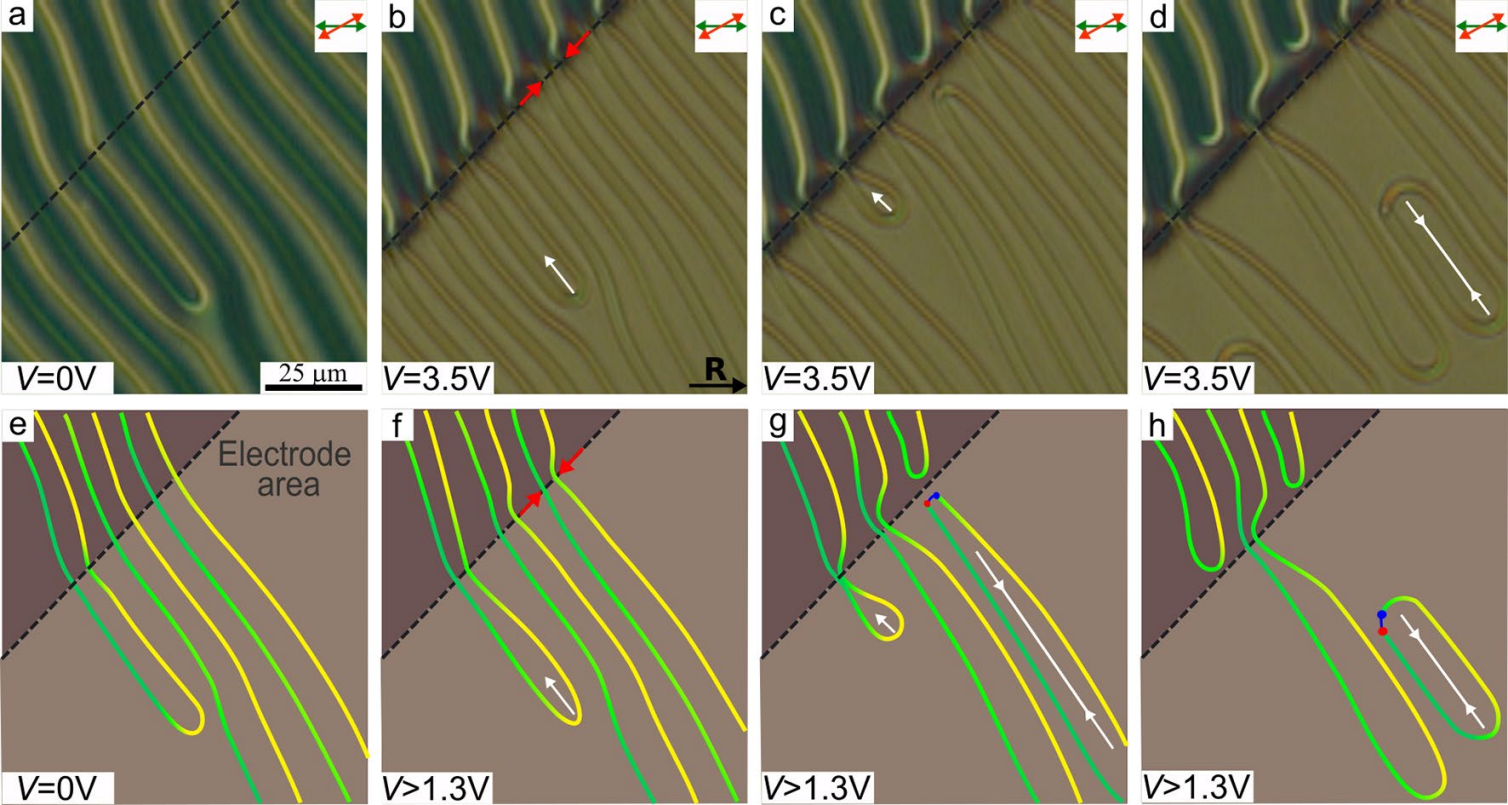
Optical textures of CLC layer when the analyzer is oriented to the polarizer at 30$^\circ$ angle in the initial state *V* = 0 V (**a**), in 0.3 s (**b**), 1.4 s (**c**), 2.9 s (**d**) after switching-on voltage 3.5 V and the corresponding schemes of defect line transformations at the electrode edge noted hereinafter by black dashed line (**e**)–(**h**). The white single and double arrows indicate the movement direction of $\hbox {U}^+$-defect and defect loop, respectively.

Electric field applied to the cell causes reorientation of LC director and, consequently, changes the overall CLC structure. The observed character of response significantly differs in two control regime: low and high voltage. Owing to the conical boundary conditions at the top substrate, the reorientation process of director under electric field is non-threshold. At low voltage, an electric field does not practically affect the director orientation near the defects since here the director tilt angles are opposite signs. Therefore, the line positions are stable. We found that such a situation is observed at $V \le 1.3$ V, when electric field causes only a color change in optical texture in the crossed polarizers (see Supplementary Figure S3). At high voltage $V>1.3$ V, $\hbox {U}^+$-defects (Fig. 4a,e) move along the defect lines toward the center of electrode area, resulting in a complete disappearance of the periodic structure (Fig. 4). As voltage increases the defect movement velocity rises from parts of microns to tens of microns per second. For instance, at $V=3.5$ V the $\hbox {U}^+$-defect velocity is about $40\,\upmu$m/s, which allows to detect visually the main stages of motion and transformation of lines (see (Suppl. Movie.1).

**Figure 5:**
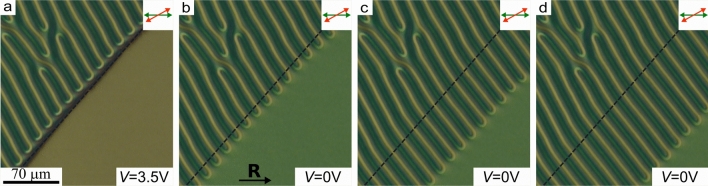
Optical textures of CLC layer with 30$^\circ$ angle between analyzer and polarizer under 3.5 V voltage (**a**) and after switching off voltage in 2.6 s (**b**), 8.4 s (**c**) and 12.1 s (**d**).

The electric field near the electrode border is non-uniform. It leads to a different transformation of defect lines near this area, where over- and under-twisted defect lines move towards each other (Fig. 4b,f). Often, this process leads to a break and pairwise connection of the defect lines at the electrode edge area (Fig. 4c,g). As a result, the $\hbox {U}^+$-defect appears from outside of the electrode edge, and simultaneously the defect line bend with the pair of singular points arises inside the electrode area (Fig. 4d,h). The total turn of the director at the bend with pair of the singular points is $-180^\circ$ ($\hbox {U}^-$-defect)^39^. The newly formed $\hbox {U}^+$-defect remains at the electrode edge outside, while the $\hbox {U}^-$-defect moves along the lines to opposite electrode edge. $\hbox {U}^-$-defect can originate by the pair of linear defects that initially contain $\hbox {U}^+$-defect. In this case, a defect loop is formed, and further it shrinks and eventually disappears under electric field^39^. Finally, all defect lines disappear in the area of electric field action. At the same time, the periodically located $\hbox {U}^+$-defects are formed on the outside of one pair of electrode edges (Fig. 5a), while the defect lines are aligned parallel to another pair of electrode borders.

### Relaxation process

**Figure 6:**
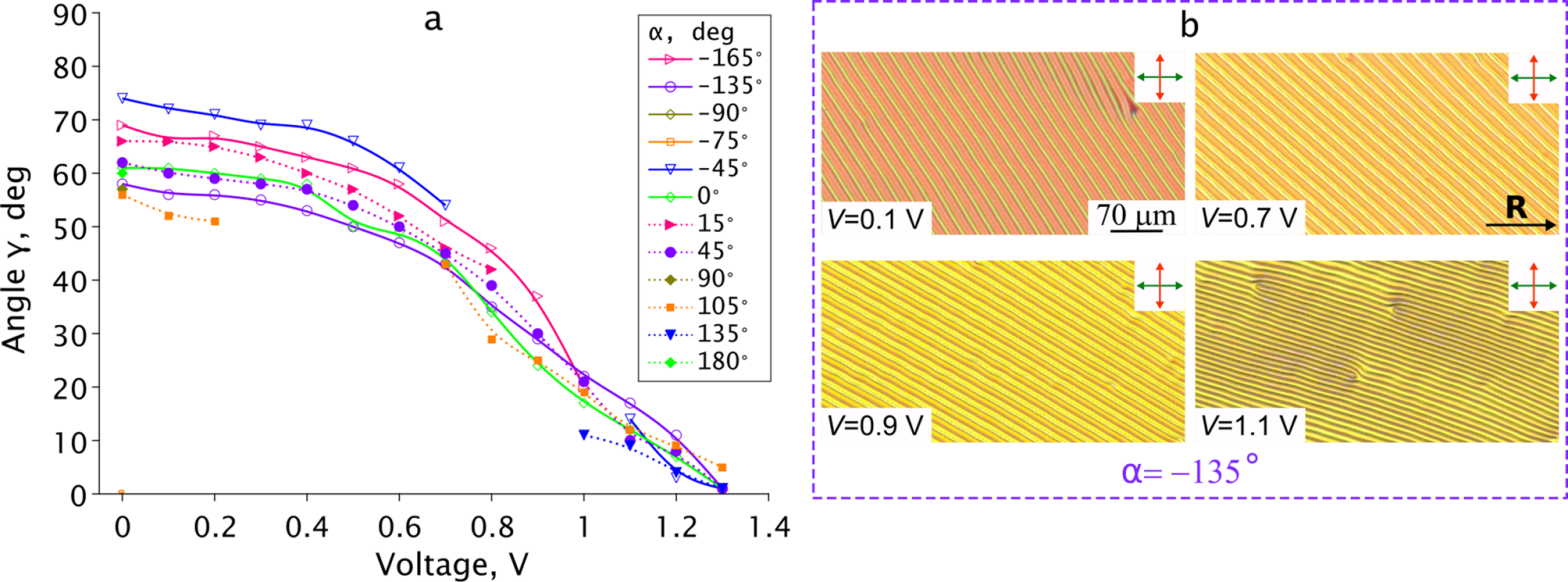
Dependence of angle $\gamma$ between the rubbing direction **R** and the defect lines appearing from different electrode sides on applied voltage (**a**). POM images of CLC layer in crossed polarizers taken after switching off indicated voltage for $\alpha =-135^\circ$ (**b**). The defect lines don’t arise for some values of angle $\alpha$ and voltages (missing points on the curves in (**a**)).

When the electric field is switched off, the U-defects located at the electrode edge move back inside electrode area (Fig. 5a–d). When $\hbox {U}^+$-defect moves towards $\hbox {U}^-$-defect, they approach each other and annihilate resulting in the pair of over- and under-twisted defect lines (Suppl. Movie.2). Thus, after switching off the voltage the initial periodic structure restores. The relaxation process can be stopped by applying the voltage $V=1.3$ V. On the one hand, it allows controlling the relationship between the areas with and without the periodic structure of linear defects. On the other hand, when the periodic structure restores $0\le V \le 1.3$ V, the direction of motion of U-defects and, consequently, the orientation of forming periodic structure depend on the value of applied voltage. Dependence of angle $\gamma$ between the rubbing direction **R** and the orientation of the formed periodic structure on applied voltage is presented in Fig. 6.

First of all, the $\gamma$ angle decreases from $55^\circ {-}75^\circ$ to $0^\circ {-}5^\circ$ under the action of electric field. Also, the exact value of $\gamma$ depends on the value of $\alpha$ angle (the orientation of electrode border to the rubbing direction, see Fig. 1a). Different $\gamma$ values were observed even at $\alpha$ of opposite sign but the same value (for example, at $V=0$ we observe $\gamma =62^\circ$ (at $\alpha =45^\circ$) and $\gamma =74^\circ$ (at $\alpha =-45^\circ$). Secondly, the highest variation in $\gamma$ angle is observed at $V\approx 0$ V (Fig. 6a). Third, the defect lines were found not to grow at some angles $\alpha$ and voltages. It is proved by the missing points in Fig. 6a (for example, see $\alpha = 105^\circ$ curve in the range $0.2<V<0.7$ V).

The dependence of $\gamma$ angle on the $\alpha$ can be explained by the influence of LC structure on the defect growing process. In the case of the twisted structure this effect occurs even when U-defects move from the parallel electrode borders but in the opposite directions. At $V<1.1$ V the period of the formed structure shows no dependence on voltage at which the defect lines arise, and the equilibrium structure period $\Lambda _{exp} = 17.5\pm 0.5\,\upmu \text {m}\cong 2.1p_0$. The formed periodic structure remains after the field switched off. If the voltage is varied during the defect lines growth, the direction of the further growth changes also in accordance with a new voltage value (see Suppl. Movie.3). Thus, the various periodic structures with assigned lines orientation in the different LC cell areas can be formed, and they will be stable when no voltage.

**Figure 7:**
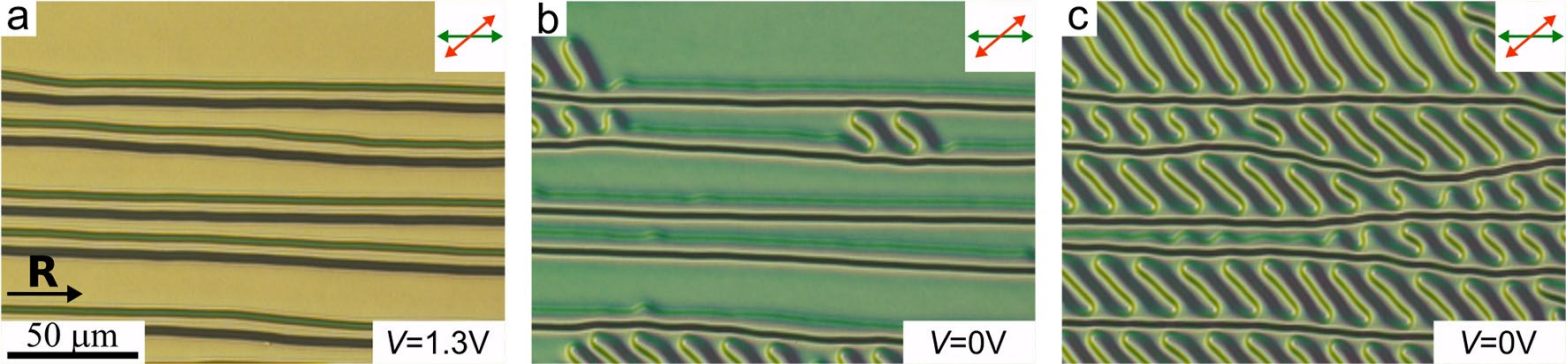
POM images of CLC layer with the defect lines formed nearly parallel to the rubbing direction **R** under $V = 1.3$ V (**a**) and in 9.2 s (**b**), 46.8 s (**c**) after switching off the field. The analyzer is oriented to the polarizer at 40$^\circ$ angle.

Under $1.1 \le V \le 1.3$ V the defect lines still grow, but their arrangement is not strictly periodic, and the average distance between them is longer than the equilibrium structure period $17.5\,\upmu$m. This effect is clearly seen at $V=1.3$ V, when the pairs of defect lines are divided by distance bigger than $\Lambda _{exp}$ (Fig. 7a). When the electric field is switched off, over-twisted defect lines curve into a series of U-defects (Fig. 7b). This process continues until the defect lines fill all the space between under-twisted lines and form quasi-periodic structure with a large number of U-defects (Fig. 7, Suppl. Movie.4). When increasing the electric field to $V>1.3$ V, these U-defects straighten and form the initial over-twisted defect lines again. In the case of the initial defect lines located along the electrode edge, the similar formation of new U-defects is observed. Due to this mechanism, it is possible to grow the periodic structure of defect lines even at the electrode edge in an arrangement with no periodic U-defects in the beginning.

**Figure 8:**
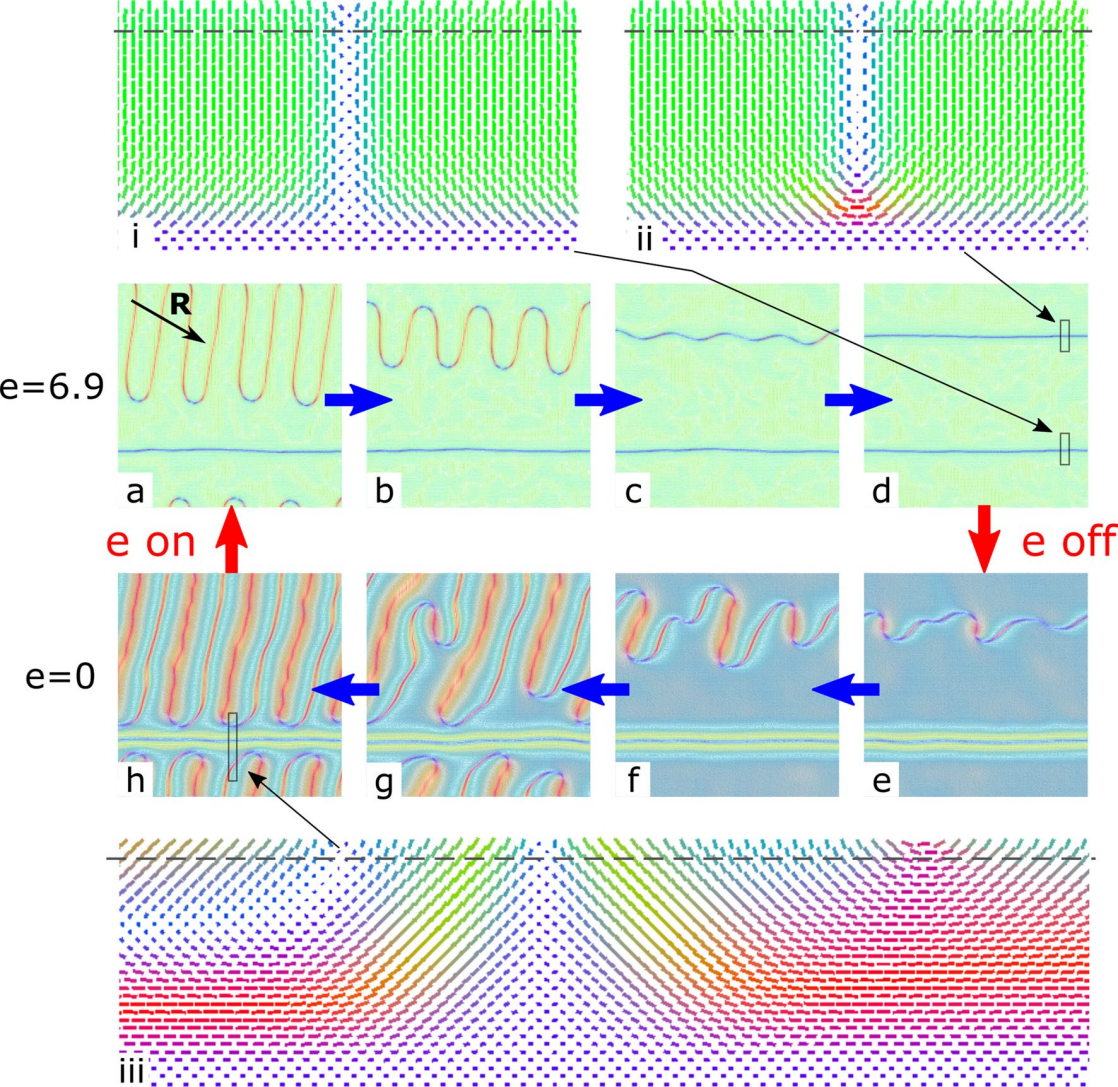
Simulations of consequent relaxation at cyclic switching the field on (**a**–**d**) and off (**e**–**h**): $e=6.9$, $10^5$ steps (**a**); $e=6.9$, $2\times 10^5$ steps (**b**); $e=6.9$, $3\times 10^5$ steps (**c**); $e=6.9$, $2\times 10^6$ steps (**d**); $e=0$, $10^5$ steps (**e**); $e=0$, $2\times 10^5$ steps (**f**); $e=0$, $5\times 10^5$ steps (**g**); $e=0$, $2\times 10^6$ steps (**h**). The colors represent the direction of director field (*x*—blue, *y*—red, *z*—green).

We have simulated this process by Monte-Carlo relaxation of the large planar system of $8d \times 8d \times d$ with periodic boundaries over *x* and *y*. It should be noted that the periodic boundary conditions of the simulations box prevented macroscopic re-orientation of the defect lines under the action of an electric field. Therefore, only a qualitative comparison between the experimental data and calculations is possible in this section. We analyzed system relaxation (i) after applying electric field along *z* axis (perpendicular to the layer) and (ii) after subsequent switching it off over multiple cycles. In agreement with the experiment, we found that the defect lines shrink only under electric fields higher than critical value $e_c\approx 3.5$. At lower electric fields, the defects remain in the initial state (Fig. 8a). At $e=6.9$, one straight defect line remains without changes, and the over-twisted defect line shortens, starting from the U-defects (Fig. 8a,b) and finally turns into straight line (Fig. 8c,d). After switching the electric field off, this defect line gradually comes back to the curved state (Fig. 8e–g) finally reaching optimal line density (Fig. 8h).

## Discussion

The periodic structures in CLC layer with conical-planar boundary conditions and their transformation under electric field have been studied experimentally and by computer simulations. Such structures present a set of alternating over- and under-twisted defect lines whose azimuthal director angle $\varphi _d$ differs by $\pi$. A smooth transition between these lines observed in the form of U-shaped curvatures in periodic structure can be identified as U-shaped defect. The orientation of the defect lines does not change under small voltage $V<1.3$ V. At higher voltage U-defects move along the defect lines.

Additionally, defect lines break near the electrode border and then form $\hbox {U}^+$/$\hbox {U}^-$ defect pair. One of these defects (outside the electrode) remains static while another one (inside the electrode area) moves along the defect lines towards the U-defect of the opposite sign. In the case of a pair of $\hbox {U}^+$/$\hbox {U}^-$ defects forming a loop, it gradually shrinks and finally disappears under the action of electric field. As a result, defect lines totally disappear in the entire electrode area after some time. Switching off voltage causes the formation and the reversed motion of U-defects accompanied by an extension of defect lines. The direction of defect line growth can be controlled by applying the voltage in the range $0\le V \le 1.3$ V. It should be noted that the period of the formed structure equals to $\Lambda _{exp}\cong 2.1p_0$ in the whole range $0\le V<1.1$ V. At $1.1 \le V<1.3$ V, the period exceeds $\Lambda _{exp}$. More complex periodic structures are formed after the action of voltage $V=1.3$ V (Fig. 7). In this case, under-twisted defect lines transform into a set of U-defects making a periodic structure between the adjacent over-twisted defect lines (Fig. 7b, c). This newly formed structure is stable at room temperature. However, it can be completely removed and the initial under-twisted defect lines can be straightened by applying a high voltage $V>1.3$ V.

Thus, the one-dimensional periodic structure oriented at various angles to the rubbing direction can be generated in CLC layer with conical-planar boundary conditions by applying of different voltages at the time of origination. Moreover, we can form the various intricate patterns of periodic structures changing the applied voltage during the formation process (Fig. 6b). The obtained results have potential in applications of such cholesterics under conical-planar boundary conditions for optical devices with the azimuthal orientation of diffraction gratings controlled by an electric field, as well as optical systems with assigned periodicity, orientation and configuration of linear defects. So, the results expand the knowledge about the possible types of orientation ordering of cholesteric liquid crystals, and they can be of interest for diffraction optics and for the identification of biological structures with analogous molecule arrangement.

## Methods

### Experimental approach

The test samples are sandwich-like cells consisting of two glass substrates with transparent ITO electrodes coated with polymer films^38,39^ (Fig. 1a). The bottom substrate was covered with the polyvinyl alcohol (PVA) (Sigma Aldrich) film and then it was unidirectionally rubbed. The top substrate was covered with the poly(isobutyl methacrylate) (PiBMA) (Sigma Aldrich) film without additional treatment after the deposition process. The spin coating method was used to deposit the polymer films on the substrates. ITO layer on the top substrate was etched as the square ($2 \times 2$ $\hbox {mm}^2$) areas oriented at the different $\alpha$ angles to the rubbing direction $\mathbf {R}$ (Fig. 1a). The values of angles formed by each of the square sides are indicated in Fig. 1a.

The CLC layer thickness $d = 5.0\,\upmu$m specified by the microspheres was measured by the interference method with spectrometer HR4000 (Ocean Optics) before the filling process. The nematic mixture LN-396 (Belarusian State Technological University) doped with cholesteryl acetate (Sigma Aldrich) was used as CLC. The cells were filled by the CLC in the mesophase at room temperature. After the filling process, the cells were kept for at least 24 h before measurements. The quantity of chiral additive was chosen to obtain the confinement ratio $d/p_0 = 0.6$. The experimental study of the sample was carried out using the polarizing optical microscope (POM) Axio Imager.A1m (Carl Zeiss). An AC voltage of 1 kHz frequency and variable amplitude were applied to the sample from signal generator AHP-3122 (Aktakom).

### Calculations of layer structure

We performed calculations of CLC structure within layer of chiral nematic. We used the extended Frank elastic continuum approach to find energy-optimal layer structures. This approach includes the effects of the director field distortion and the formation of defects:

$$\begin{aligned} F&=\int \limits _V\left[ \frac{K_{11}}{2}\,(\mathrm {div}{\mathbf {n}})^2 +\frac{K_{22}}{2}\,({\mathbf {n}}\cdot \mathrm {rot}{\mathbf {n}}+q_0)^2 + \frac{K_{33}}{2}\,[{\mathbf {n}}\times \mathrm {rot}{\mathbf {n}}]^2 + \varepsilon _0\Delta \varepsilon ({\mathbf {E}}^2-({\mathbf {E}}\cdot {\mathbf {n}})^2)\right] dV\\&\quad + \frac{W}{2}\int \limits _\Omega \left[ 1-\cos ^2(\theta _{{\mathbf {n}}{\mathbf {k}}} -\theta _0)\right] d\Omega +F_{def}, \end{aligned}$$

where $K_{11}$, $K_{22}$ and $K_{33}$ are the splay, twist and bend elasticity constants, respectively, $q_0=2\pi /p_0$, *W* is the surface anchoring energy density, $\theta _{nk}$ is the tilt angle of local director ${\mathbf {n}}$ from the surface plane, $\theta _0$ is the preferred tilt angle, and $F_{def}$ is the energy of defects calculated by the summation of the point and linear defect energies (see the details in ref.^44^). The ratio between elasticity constants was set to $K_{11}:K_{22}:K_{33} = 1:0.51:1.31$ to simulate the studied cholesteric liquid crystal mixture. To take into account potential formation of the disclination lines with core, its linear energy density was set to $f_{core}^{line} = K_{11}$. The bottom surface was set with strong planar aligned boundaries characterized by the dimensionless anchoring strength $\mu _1 = W_1d/K_{11} = 1000$ and $\theta _0=0$, where *d* is the thickness of the layer. The top surface had weak conical boundary conditions ($\mu _2 = W_2d/K_{11} = 40$, $\theta _0=50^\circ$). The equilibrium characteristic ratio $d/p_0=0.6$ was set according to the experimental data. To simulate thin layer, we used cuboid simulation box with periodic boundary conditions over two dimensions (namely, *x* and *y*). We varied the first dimension $L_x$ from 2*d* to 4*d* with 0.1*d* step to find energy-optimal stripped structure period. The volume was rendered in a lattice from $32\times 4\times 16$ to $64\times 4\times 16$, correspondingly. The second dimension of the simulations box was fixed at $L_y=0.25d$ to constrain a possible direction of strips parallel to *y*. For each simulation box size, we varied the direction of easy axis on bottom plane from 0 to $180^\circ$ with $5^\circ$ degree step to determine the energy-optimal mutual orientation of defect lines and rubbing direction. We used Monte-Carlo annealing optimization with 16 independent runs for each setup to find the energy-optimal structures. To simulate electrically-induced transformation of LC structure, we applied the Monte-Carlo relaxation after switching the electric field on or off. The data shown in dimensionless electric field were calculated as $e=|{\mathbf {E}}|{d}\left( \varepsilon _0\Delta \varepsilon /K_{11}\right) ^{1/2}$.

## Supplementary Information

Supplementary Video 1.

Supplementary Video 2.

Supplementary Video 3.

Supplementary Video 4.

Supplementary Information.

## Data Availability

All data generated and analyzed during this study are available upon request.
