# Supplementary material for: Electrically turning periodic structures in cholesteric layer with conical–planar boundary conditions
Source: Sci Rep. 2021 Apr 16;11:8409. doi: 10.1038/s41598-021-87854-z (PMC8052423; doi:10.1038/s41598-021-87854-z)
Supplement: Supplementary file 5 — Supplementary Information. [file 41598_2021_87854_MOESM5_ESM.pdf]

Supplementary Information

Electrically Turning Periodic Structures in Cholesteric Layer with Conical-Planar Boundary Conditions

Oxana Prishchepa[1,*], Mikhail Krakhalev[1,2], Vladimir Rudyak[3], Vitaly Sutormin[1,2], and Victor Zyryanov[1]

[1] Kirensky Institute of Physics, Federal Research Center KSC SB RAS, Krasnoyarsk 660036, Russia

[2] Institute of Engineering Physics and Radio Electronics, Siberian Federal University, Krasnoyarsk 660041, Russia

[3] Faculty of Physics, Lomonosov Moscow State University, Moscow, 119991, Russia

[*] Corresponding author: O. Prishchepa, e-mail: p_oksana@iph.krasn.ru

Supplementary Figure S1

Supplementary Figure S2

Supplementary Figure S3

Supplementary: Movie 1, Movie 2, Movie 3, Movie 4.

Supplementary Figure S1| POM image of CLC layer under planar-conical boundary conditions at initial state before application of voltage (a). CLC structure is randomly distributed areas where the strips orient at various angles γ to the rubbing direction. The thickness of the CLC layer d is 5 μm. The ratio d/p_0 is 0.6; the polariser's directions are noted by the double arrows, the single arrow indicates the rubbing direction **R**. POM photos are taken in white light. The enlarged area with U-defects marked by the black frame (b).

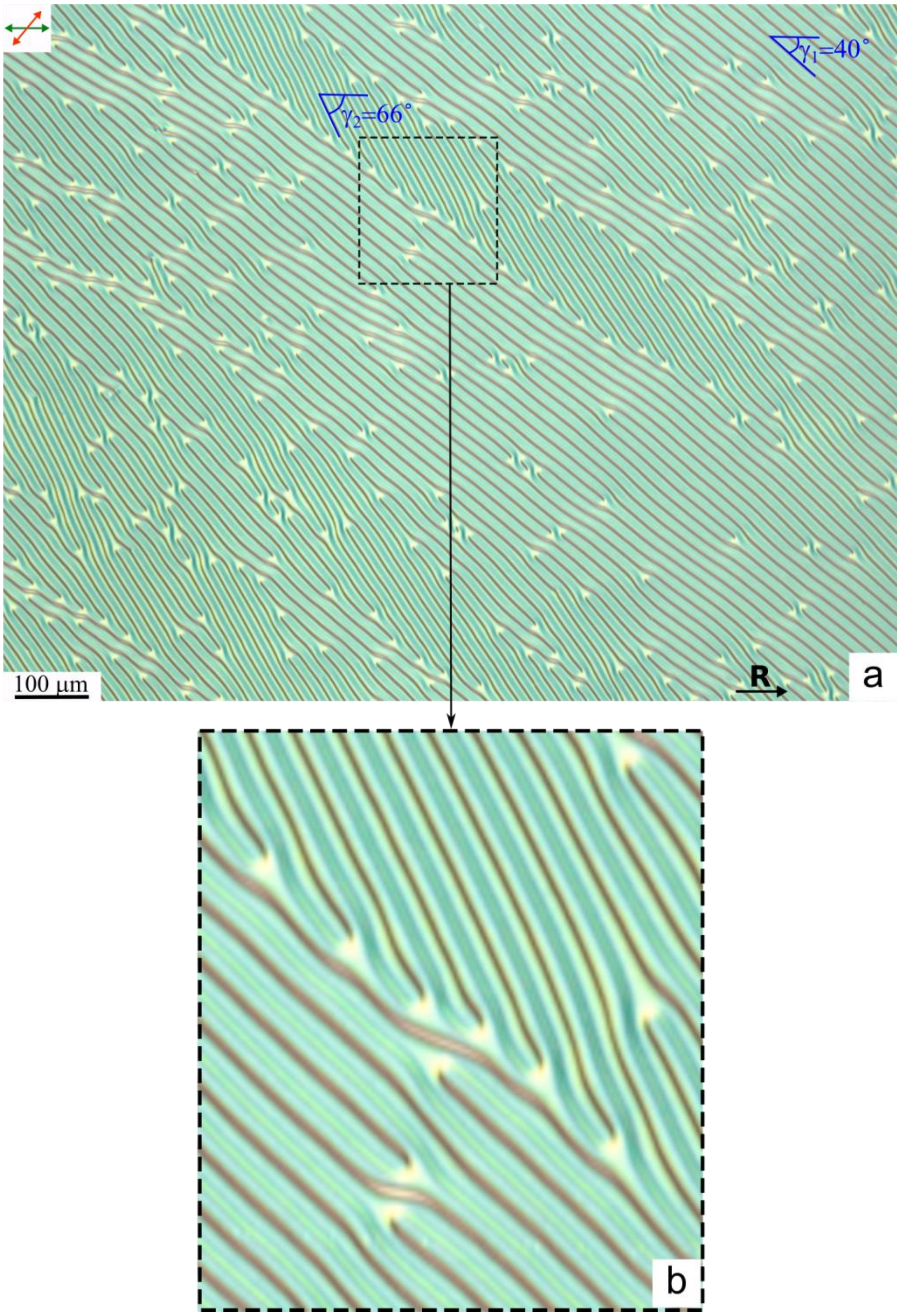

Supplementary Figure S2| Director distribution for energy-optimal simulated structure in cross sections of cholesteric layer perpendicular to the strip lines at various levels of *d* (0, 0.2, 0.4, 0.6, 0.8 and 1.0). The director vectors are colored in correspondence with their direction (x - blue, y - red, z - green). Black lines show the locally averaged orientation of CLC director.

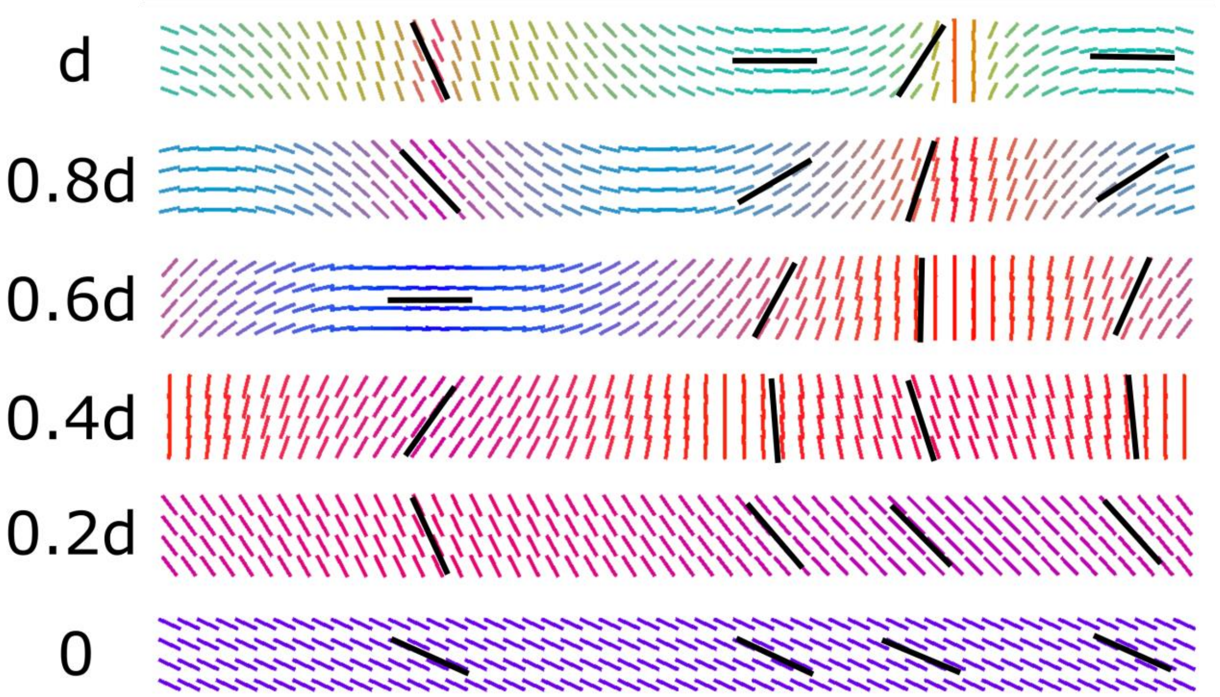

Supplementary Figure S3| Optical textures of CLC layer under planar-conical boundary conditions in crossed polarizers under 0V (a), 0.8V (b), 1.0V (c), 1.2V (d). The initial structure was formed at voltage 0.8 V. Cross-section of the director field at V =0V (e) and at V < 1.3V (f). The angle between the director at the bottom substrate and over-/under-twisted line is 35°.

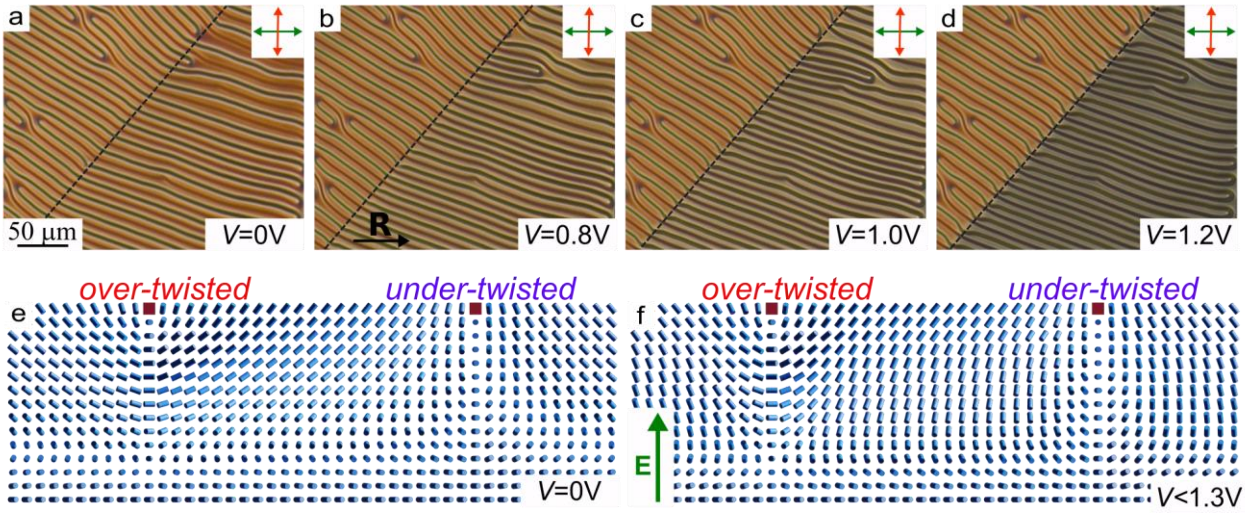

The reorientation process of director under electric field is nonthreshold due to the conical boundary conditions at the top substrate. An electric field doesn't practically change the director orientation near the defects since here the director tilt angles are opposite signs (Figures S3f). Therefore, the line positions are stable at V<1.3 V.

Supplementary Movie 1| Movement of U-defects and defect lines at switching on/off voltage.

Supplementary Movie 2| Coupling of U+ and U- defects.

Supplementary Movie 3| Electrically controlled direction of defect line growth.

Supplementary Movie 4| Formation of quasi-periodic structure.
